# Enhancement of Mechanical Properties of Benign Polyvinyl Alcohol/Agar Hydrogel by Crosslinking Tannic Acid and Applying Multiple Freeze/Thaw Cycles

**DOI:** 10.3390/gels10080527

**Published:** 2024-08-12

**Authors:** Moustapha Mohamed Mahamoud, Tadesse Mekonnin Ketema, Yutaka Kuwahara, Makoto Takafuji

**Affiliations:** 1 Faculty of Advanced Science and Technology, Kumamoto University, Kumamoto City 860-8555, Japan; harroun92@gmail.com (M.M.M.); tmketema@gmail.com (T.M.K.); kuwahara@kumamoto-u.ac.jp (Y.K.); 2 International Research Organization for Advanced Science and Technology (IROAST), Kumamoto University, Kumamoto City 860-8555, Japan

**Keywords:** polyvinyl alcohol, agar, tannic acid, tensile strength, swelling ratio, freezing, thawing, crystallinity, adhesion

## Abstract

Hydrogels composed of natural and synthetic polymers have considerable potential for use in diverse areas such as biomedical applications and water purification. This is primarily because of their biocompatibility, biodegradability, and low toxicity. The widespread usage of composite hydrogels is hindered by a lack of simultaneous properties, such as high strength and low swelling rate. Herein, we report the preparation of novel hydrogels composed of polyvinyl alcohol (PVA)–intercalated agar polymer networks physically crosslinked with tannic acid. The hydrogel was subjected to multiple freeze/thaw (F/T) cycles (1, 3, and 5), and it was found to exhibit the highest strength after 5 F/T cycles. After 1 F/T cycle, the tensile strength of the composite hydrogel reached 1.56 MPa with a 1.0 wt% crosslinker, whereas after 5 F/T cycles, it increased to 3.77 MPa with a reduced amount (0.75 wt%) of the crosslinker. In addition, the swelling ability decreased upon increasing the crosslinker content and number of F/T cycles. Furthermore, the hydrogel demonstrated excellent water retention and a strong ability to adhere to different substrates. We have successfully implemented an innovative approach to improve the mechanical properties of PVA-based hydrogels by combining the use of tannic acid as a cross-linking agent and multiple F/T cycles. The developed hydrogels are expected to facilitate new developments in hydrogel technology, thus impacting diverse fields such as biomedical (wound dressing and artificial cartilage).

## 1. Introduction

A hydrogel is a three-dimensional polymeric network that is capable of retaining a large amount of water. It has attracted considerable attention in various fields such as biomedicine, water purification, agriculture, and energy. Despite having multiple advantageous properties, most hydrogels are limited by low mechanical strength and high swelling rates in practical applications. Accordingly, hetero-network hydrogels composed of two contrasting and linked polymer networks have been developed into a single composite gel in the effort to enhance strength and eventually reduce excessive water absorption. In this context, polyvinyl alcohol (PVA) has received considerable research attention because of its chemical stability, low toxicity, strong biocompatibility, biological degradation resistance, high water absorption capacity, and simple processing. Several researchers have attempted to fabricate composites of synthetic materials, such as PVA and biopolymers/nanoparticles, which demonstrate suitable properties for new and interesting applications. Stauffer and Peppas [1] developed a technique involving freezing/thawing of a PVA hydrogel (composed of PVA in water at 10–15 wt% by heating in an oven set to 90 °C for no more than 6 h) and reported some distinctive characteristics of PVA. They found that the gel retained the same size and shape at room temperature, but it could be stretched up to five to six times larger than before. This shows that the PVA hydrogel is rubbery, flexible, and has good mechanical strength. Its properties may change depending on the molecular weight (Mw) of the polymer, concentration of the aqueous solution, freezing temperature and time, and number of freeze/thaw (F/T) cycles [1]. Lozinsky et al. [2] used gel chromatography to demonstrate that the molecular weight distribution of PVA was unchanged before and after freezing and thawing. Lozinsky et al. [2] investigated PVA gels at high temperatures and found that the PVA crystallites melted at approximately 70–80 °C and rapidly decomposed in boiling water. Watase and Nishinari performed X-ray diffraction (XRD) analysis, revealing a halo effect at 26 °C [3]. This indicates that PVA has an ordered microcrystalline structure. Yokoyama et al. [4] employed XRD to show that the number of crystallites increases with the number of F/T cycles. Fumio et al. [5] observed that the amount of physical crosslinking increased as the number of F/T cycles increased. They also measured the mechanical storage modulus to determine the elasticity of PVA hydrogels. The elasticity was found to depend on the number of F/T cycles. As the number of F/T cycles was increased, the increase in rigidity was attributed to an increase in density and chain entanglement. Furthermore, they investigated the changes in the storage modulus, loss modulus, and loss tangent with temperature. As the temperature increased, the moduli decreased. Only a slight decrease was observed in the moduli when the temperature was increased in the range of 40–50 °C. Subsequently, the moduli decreased rapidly. Initially, the loss tangent slightly decreased, but increased when the temperature reached 50 °C. Numerous scientists have studied PVA with biopolymers and crosslinkers and reported favorable results. Locarno et al. [6] introduced a novel double-network hydrogel based on PVA crosslinked with glutaraldehyde, enhancing its mechanical properties with self-assembling phenylalanine (Phe) derivatives for 3D dosimetry applications. The combination of biopolymers with synthetic polymers in hydrogels can yield materials with improved biocompatibility, bio-functionality, and potentially better environmental sustainability. Sabzi et al. [7] developed a PVA/agar double network (DN) hydrogel, wherein both networks were physically crosslinked (via hydrogen bonds). They prepared the DN hydrogel in a simple one-pot method (with all the reactants in one pot) with heating and cooling. The DN hydrogel exhibited a high elastic modulus of up to 2200 kPa, toughness of up to 2111 kJ/m^3^, and the ability to self-heal after a cut, while autonomously regaining up to 67% of its original strength after 10 min. Samadi et al. [8] developed completely physically crosslinked DN hydrogels of PVA/agar by adding nanocomposite graphene (G) as a third network. They found that the addition of graphene nanoplatelets into agar/PVA DN hydrogel enhanced the mechanical properties, as agar/PVA-G triple network nanocomposite hydrogels achieved excellent strength (1157 kPa), ~500% strain, and high toughness (2610 kJ/m^3^). Shao et al. [9] reported a completely physically crosslinked PVA/agarose DN. The study and method of preparation were practically similar to that of Sabzi et al.; however, instead of agar, they used agarose, which is one of the two principal components of agar; it was purified from agar by removing agaropectin, the other component of agar. When the agarose content was 5%, the hydrogel had the highest compressive strength (3.66 MPa), which was eight times higher than that of pure PVA hydrogels. Pourjavadi et al. [10] designed a DN hydrogel based on chitosan and PVA, demonstrating high mechanical performance; they used cationic polymer chitosan as the first network and sodium phosphate as the physical crosslinking agent. Neutral PVA, as the second network, was crosslinked through F/T cycles. PVA and chitosan are not completely miscible owing to their weak interactions. Therefore, honey was used as a unique plasticizer to enhance the interactions between PVA and chitosan by forming hydrogen bonds. The chitosan–PVA DN hydrogel with honey as the plasticizer and 50% sodium phosphate exhibited a considerable improvement in mechanical properties (10.6 MPa with an elongation of 390%).

Herein, we propose the design of a non-toxic hydrogel composed of a PVA–agar matrix loaded with tannic acid (TA). PVA is a thermoplastic polymer obtained from the hydrolysis of polyvinyl acetate [11]; agar is a mixture of agarose polysaccharides and smaller polysaccharides called agaropectin; TA is a naturally occurring plant polyphenol that is found in nearly all aerial plant tissues [12,13]. The structure is composed of five digallic acid units connected to a central glucose core [14]. TA possesses a high concentration of carbonyl groups, which enables it to participate in many interactions such as hydrogen bonding, ionic bonding, and hydrophobic interactions with several small molecules and polymers.

The polymers are physically crosslinked via interactions between the hydroxyl groups of PVA, agar, and TA [15]. The process of fabricating the hydrogels composed of PVA and agar crosslinked with TA (PA-Tx (X = concentration of TA (wt%)) involves heating and repeated freezing/thawing. The study focuses on integrating crucial properties into a single hydrogel. The obtained hydrogels are evaluated in terms of tensile strength [16], water-swelling ability, water-retaining capacity, and adhesion to glass, aluminium, and natural rubber.

## 2. Results and Discussion

### 2.1. Fabrication of PVA Composite Hydrogel

The PA-Tx hydrogel was prepared by heating, cooling, and freezing/thawing. An aqueous solution of PVA was mixed with different amounts of TA and then heated at 95 °C for 3 h and mechanically stirred. Subsequently, the agar polymer was added to the mixed solution, and the entire system was heated at the same temperature for 1 h. The solution was then cooled to room temperature (25 °C). Finally, the solution was frozen at −15 °C for 24 h and thawed for 4 h; F/T cycling was conducted 1, 3, and 5 times.

**Figure 1:**
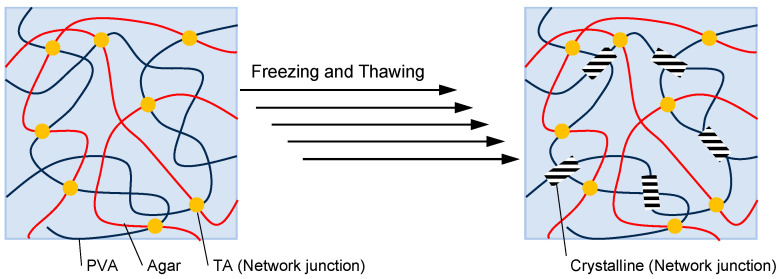
Schematic of the preparation of PA-Tx hydrogels through freezing/thawing. The arrow indicates the freezing and thawing cycle.

**Scheme 1:**
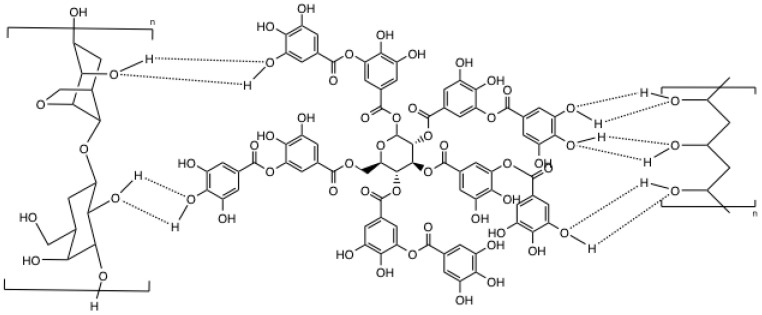
Illustration of PA-Tx hydrogel formation.

During the freezing step (Figure 1), phase separation becomes stronger owing to the ice crystals that bring the PVA and agar chains closer together [4]. This causes the PVA-rich phase to separate from the aqueous phase. As the F/T cycles proceed, the polymer-rich areas acquire additional chains, which are brought closer together, thus facilitating hydrogen bonding and crystallization [17]. Repeated F/T cycling transfers additional polymer chains to densely populated areas, bringing them closer for easier hydrogen bonding and crystallization [5,18]. Tannic acid plays a significant role in the crosslinking mechanism of polyvinyl alcohol of PVA and agar due to its multiple phenolic hydroxyl groups. In this work, tannic acid acts as a crosslinker since it contains a large amount of hydroxyl group (OH) that can bond with the OH groups of PVA and agar (Scheme 1). Thus, this results in the formation of physical crosslinking, specifically hydrogen bonding. Furthermore, TA crosslinking strengthens the hydrogel by multiplying the crosslinking points, and the chains in the network system are highly interconnected (Scheme 1) owing to the dual crosslinking: crystalline formation (PVA) and intermolecular hydrogen bonding (TA with PVA and agar).

### 2.2. Effects of Freezing Temperature, Freezing Time, and Number of F/T Cycles on the Mechanical Strength of PVA-Based Hydrogels

**Figure 2:**
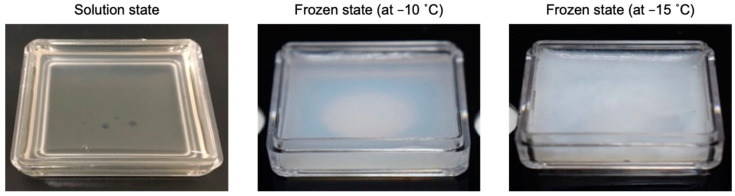
Photographs of solution state (**left**) and frozen states (**middle** and **right**) after induction at different freezing temperatures (1 freeze–thaw (F/T) cycle).

**Figure 3:**
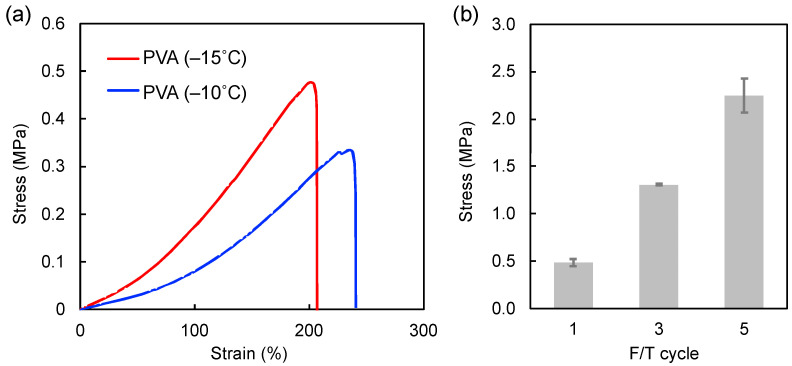
(**a**) Tensile strength of PVA hydrogels (15 wt%) prepared with 1-F/T cycle at freezing temperatures of −15 and −10 °C, and (**b**) tensile stress at breaking point as a function of F/T cycles.

Several experiments were conducted to determine the appropriate freezing temperature for hydrogel gelation. A hydrogel was made from PVA. According to Locarno et al. [19], the hydrogel’s crosslinking density and mechanical strength increased as the degree of hydrolysis increased. The PVA used in the present study had a degree of hydrolysis of 99–99.8 mol% and an average Mw of 145,000, with 15 wt% concentration. Gelation occurred at both −10 and −15 °C (Figure 2). Additionally, the hydrogel strength (Figure 3) was verified at −10 and −15 °C; the hydrogel prepared at a lower temperature (−15 °C) exhibited higher strength. Therefore, the appropriate temperature for the subsequent experimental steps was set to −15 °C.

Regarding the freezing time, Yokoyama et al. [4] reported that −20 °C for 8 to 12 h is widely adopted for the freezing step. In another study, Stauffer and Peppas [1] observed that longer freezing times increased the crystallinity of the hydrogels in addition to enhancing their strength and stiffness. Regarding the F/T cycles, Yokoyama et al. [4] showed that additional PVA chains are implicated in phase separation, crystallization, and gelation with the increasing number of F/T cycles because the chains are driven into polymer-rich areas. Consequently, the hydrogel stability, mechanical strength, elasticity, and resistance to water are enhanced. In the F/T process, crystallization and phase separation are balanced. Furthermore, crystallization and phase separation were previously observed to occur more frequently in the early cycles and at least in the sixth cycle [4,20]. Gelation can possibly occur after only one cycle under particular conditions (high Mw and PVA content). Although the gels may lose a substantial amount of mass when submerged in water, this is to be expected, considering the early age and development of the network. This loss is caused by the breakdown of the weak connections of the network or the unwinding of un-crosslinked strands. Furthermore, the high adhesion capacity of the gels at low F/T cycles decreases with an increasing number of cycles. PVA hydrogel with 15 wt% concentration was subjected to multiple F/T cycles (1, 3, and 5 times) at −15 °C, and the highest strength was observed at 5 F/T (Figure 3b).

### 2.3. Fourier-Transform Infrared Analysis

**Figure 4:**
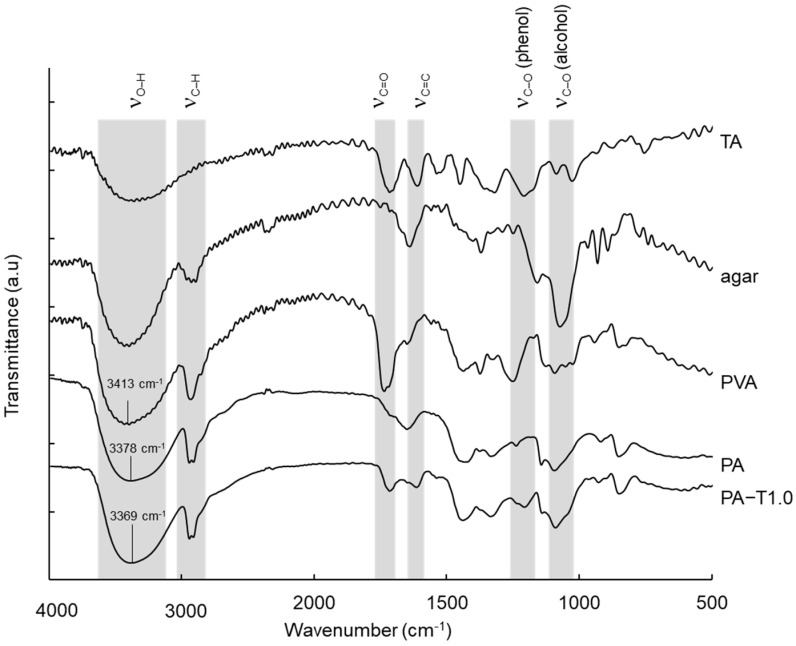
Fourier-transform infrared spectra of hydrogels composed of TA, agar, PVA, PA, and PA-T1.0 (refer to Table 1).

The solid states of PVA, agar, TA and vacuum-dried hydrogel composed of PVA and agar (PA), and PA–T1.0 hydrogel were characterized using Fourier-transform infrared (FT-IR) spectroscopy, and the results are shown in Figure 4. The stretching vibration absorption peak of O–H or hydrogen bonds caused broad absorption bands between 3000 and 3750 cm^−1^, whereas the absorption peaks at 2923 and 1087 cm^−1^ were attributed to C–H and C–O stretching vibrations, respectively. TA was present between PVA and agar at 1710 (C=O), 1207 (C–O), and 1610 cm^−1^ (C=C). This indicated that TA was successfully loaded between PVA and agar. Furthermore, the OH stretching peak shifted from 3413 to 3369 cm^−1^, indicating that PVA, agar, and TA formed strong hydrogen bonds.

### 2.4. Mechanical Strength of the Hydrogel

**Figure 5:**
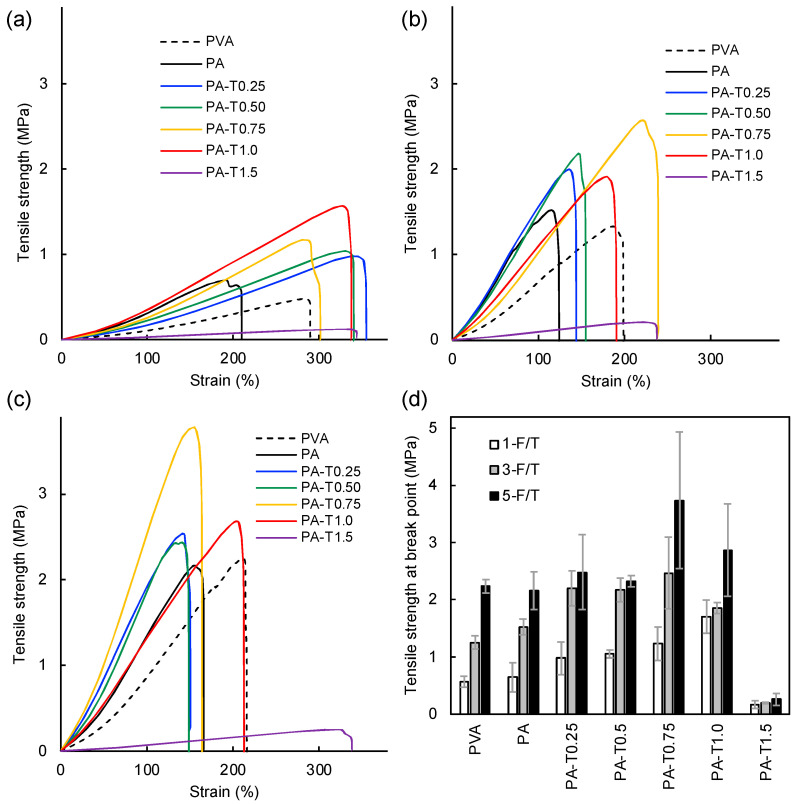
Tensile strength–strain curves of composite hydrogels (PVA, PA, and PA-T1.0 (refer to Table 2) at (**a**) 1-F/T, (**b**) 3-F/T, and (**c**) 5-F/T and (**d**) average of tensile strength at break point with error bar (standard deviation).

Tensile strength is a crucial property of hydrogels for various applications. Figure 5 shows the tensile stress and strain graphs of the hydrogel after multiple F/T cycles (1, 3, and 5). The tensile strength of the hydrogels increased with the increasing number of F/T cycles. Based on these results, the PA-Tx composite hydrogels reached their maximum strength after 5 F/T cycles, with a 0.75 wt% crosslinker. The addition of TA as a crosslinker to the composite increased the strength to a certain extent; particularly, it resulted in additional crosslinking points. In contrast, when the hydrogel was already subjected to multiple F/T cycles (5 times), a lower amount of crosslinker was required to obtain the highest possible strength. For example, at 3 and 5 F/T, only 0.75 wt% crosslinker is required to obtain the highest tensile strength, and this is a smaller amount than that at 1 F/T (1.0 wt%). Therefore, the PVA-based hydrogel prepared with the F/T process involves the growth of crystallites in the PVA hydrogel network that predominantly occupy the hydrogel matrix; the more the hydrogel is subjected to multiple F/T processes, the less it requires a crosslinker to attain the maximum possible strength.

### 2.5. Crystallization Analysis

In the pure PVA hydrogel, a sharp crystalline reflection with extremely high intensity was observed at approximately 19.2° [21]. The crystallinity of the PVA hydrogel markedly increased when the number of F/T cycles was increased from 1 to 3, and then it slightly increased from 3 to 5 F/T. PVA has been extensively studied and is known to be crystalline owing to the strong intermolecular contact between the PVA chains via hydrogen bonding. The number of PVA chains grouped together influences the degree of PVA crystallinity [22]. The increase in the degree of crystallinity with reference to the number of F/T cycles is listed in Table 2. These findings agree with those reported by Ricciardi et al. [23], who employed XRD and differential scanning calorimetry. They reported that freezing and thawing increased the crystallinity of PVA-based hydrogels. Furthermore, crystallization and phase separation occurred, as reported by Holloway et al. [20].

**Figure 6:**
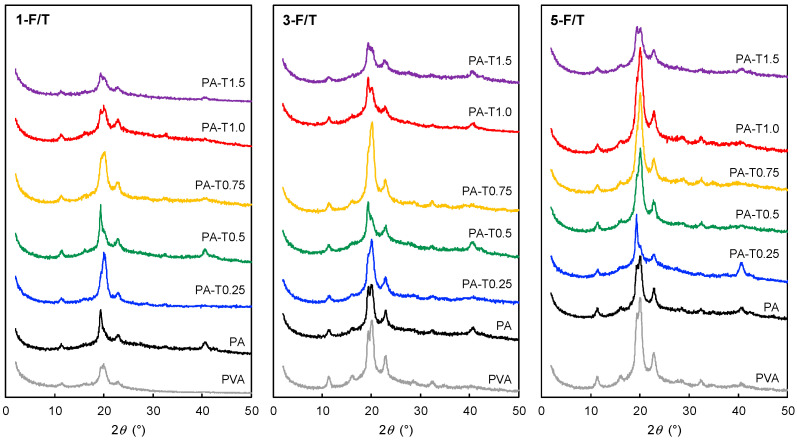
X-ray diffraction patterns of PVA, PA, and PA-Tx hydrogels prepared with different F/T cycles.

We evaluated PVA, PA, and PA-Tx for different F/T cycles (1, 3, and 5) (Figure 6). Further addition of TA could possibly reduce crystallinity and influence the overall mechanical properties of the hydrogel. In this study, crystallinity increased when up to 0.75 wt% of TA was added; however, beyond the threshold amount (0.75 wt%), the effectiveness of the crosslinker and the extent of crystallization decreased considerably.

### 2.6. Swelling Ratio

**Figure 7:**
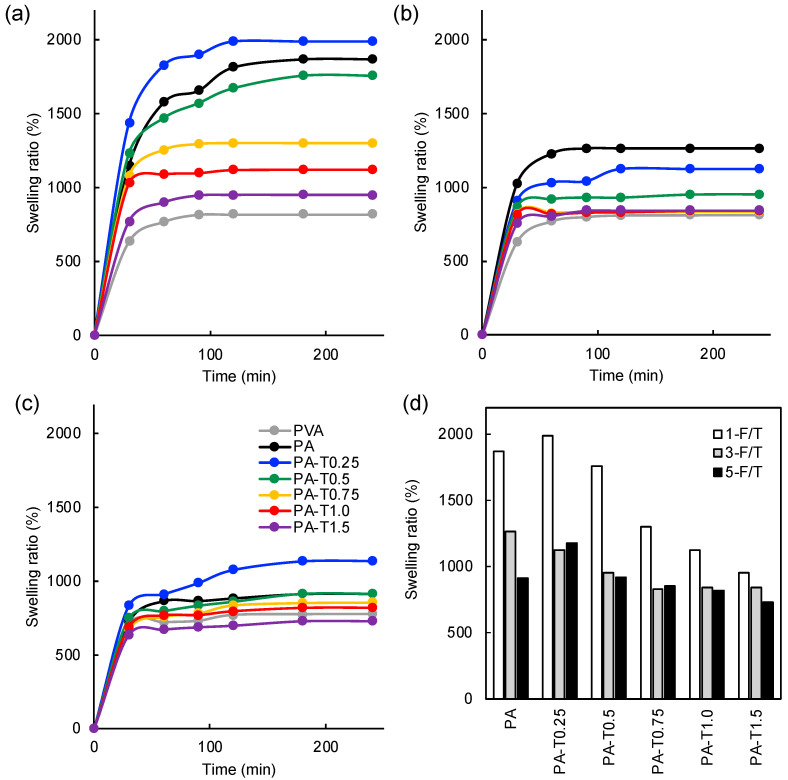
Swelling ratios of PVA, PA, and PA-Tx after (**a**) 1-F/T, (**b**) 3-F/T, and (**c**) 5-F/T cycles, and (**d**) effect of TA on the swelling ratio during multiple F/T cycles.

The swelling ratio of hydrogels is a critical feature for various applications. Hydrogels with high water-absorbing capacity are useful for the diffusion of nutrients, bioactive molecules, and waste [24]. Although high water absorption is important, it reduces the strength of hydrogels. As shown in Figure 7a–c, the hydrogels tended to swell in the first 30–120 min, after which they stabilized and reached an equilibrium state. The swelling ratios of PVA, PA, and PA-Tx hydrogels after 1 F/T were markedly higher than those after 3 and 5 F/T. Figure 7d shows that an increased number of F/T cycles renders the hydrogel less capable of expanding or swelling. Pizano et al. [25] demonstrated that a lower freezing temperature or repeated freezing steps for PVA–chitosan hydrogels resulted in higher porosity but smaller pores. Therefore, a longer freezing duration led to a lower swelling rate because of the increase in porosity. The observed swelling rates are consistent with the findings of Pizano et al. [25], and this feature requires consideration because the hydrogel would not absorb natural fluids in biomedical applications and may fit the condition to be used as artificial cartilage. The addition of TA increased the crosslinking point and densified the hydrogel network, thereby reducing water absorption (Figure 7a–c).

### 2.7. Determination of Water-Retaining Capacity

The ability of hydrogels to hold native water makes them valuable for applications, such as medical wound dressings, medication delivery systems, and agricultural soil moisture management. The centrifugation method (high-speed rotation) was selected to evaluate how efficient the polymer network can hold its existing water in order to fit in the context of implanted artificial cartilage. It is important to understand the hydrogel’s water retention behavior under long exposure to motion conditions.

**Figure 8:**
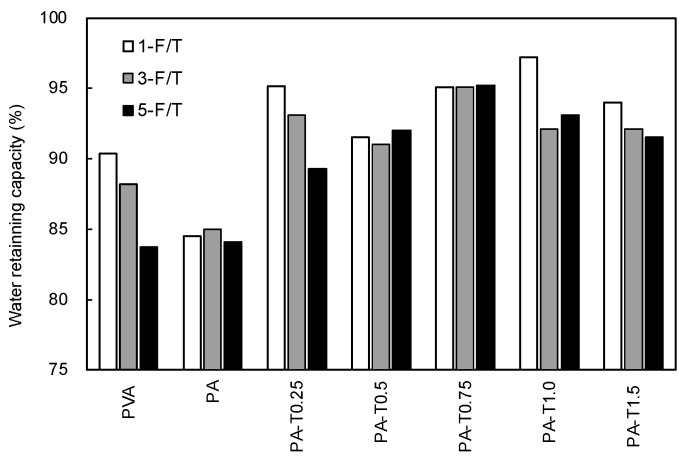
Water-retaining capacity of PVA, PA, and PA-Tx for 1, 3, and 5 F/T cycles.

In this study, hydrogels were centrifuged at 4800 rpm for 10, 20, and 30 min at 40 °C. As shown in Figure 8, most hydrogels subjected to multiple F/T cycles (1, 3, and 5 times) retained more than 80% water. The addition of TA resulted in an extremely low water release compared to the hydrogels without the crosslinker. In terms of multiple F/T cycles, Pizano et al. [25] confirmed that the hydrogel became exceedingly porous, and the water retention changed appreciably with repeated F/T cycles.

### 2.8. Adhesion of PA-Tx Composite Hydrogel

**Figure 9:**
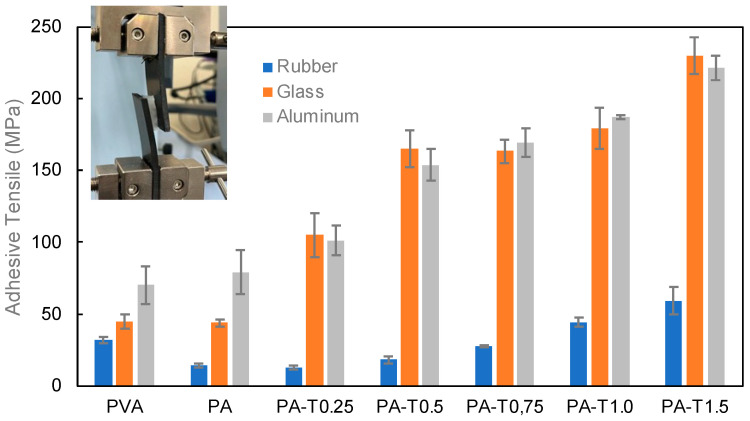
Effect of TA on the adhesion of the PA-Tx hydrogels (1 F/T) with natural rubber, glass, and aluminium substrates.

A hydrogel with high mechanical strength, low swelling ratio, and good adhesion to different substrates is considered a multipurpose hydrogel and can be used in various fields. The composite PA crosslinked with TA has the potential to bind to organic and inorganic surfaces. TA links to PVA and agar through hydrogen bonding, facilitated by its abundant pyrogallol and catechol groups. These catechol groups have the unique ability to adhere to both organic and inorganic surfaces. They can form reversible non-covalent interactions, such as hydrogen bonds and van der Waals forces, or irreversible covalent bonds, providing strong and durable adhesion. Consequently, hydrogels that incorporate catechol groups exhibit highly adhesive properties, making them suitable for a variety of applications requiring robust and versatile adhesion [22]. We examined PVA hydrogels for 1, 3, and 5 F/T cycles and found that the hydrogels demonstrated higher adhesion at 1 and 3 F/T. This is because the more we subjected the hydrogel to the F/T process, the fewer pyrogallol/catechol groups were available to bind to the substrate. To investigate the effects of TA, we studied only the 1 F/T hydrogels. As shown in Figure 9, the addition of TA gradually increased the adhesion of the hydrogel material to the natural rubber, glass, and aluminium substrates. The adhesive strength is proportional to the amount of TA, and the higher the amount of crosslinker, the higher the adhesion. Table 3 lists various PVA-based hydrogels and other hydrogel composite reported in the literature and the hydrogel developed in this study with reference to their adhesion on various substrates.

## 3. Conclusions

We prepared an environmentally friendly hydrogel composed of a biodegradable material with the following merits.

- Enhanced mechanical strength (up to 3.77 MPa)
- Reduced water absorption (~730%)
- Less water release (ability to hold it is original water content for more than 80%)
- High adhesion on glass (232 KPa), aluminium (227 KPa), and rubber (60 KPa)

We also demonstrated a strategy to increase the mechanical strength through multiple F/T cycles (up to 5) and the addition of a specific amount of TA (0.75–1.0 wt%) as a crosslinker. The enhancement in the mechanical properties was confirmed based on the increasing crystallinity of the hydrogel when the number of F/T cycles was increased from 1 to 5. Moreover, the mechanical strength associated with the hydrogel is inversely related to its flexibility. TA increases the mechanical strength, decreases the excessive swelling rate, and promotes the adherence of the hydrogel to various substrates. According to Cheng et al., the combination of PVA and agar loaded with tannic acid presented promising characteristics in terms of hemostatic, antibacterial activity, cytocompatibility, and rapid wound healing [34]. Finally, the composite hydrogel developed in this study can effectively be applied in crucial areas such as artificial cartilage.

## 4. Materials and Methods

### 4.1. Materials

PVA (Mowiol^®^ 28-99, Mw = ~145,000 g/mol, and degree of hydrolysis >99%) was obtained from Sigma-Aldrich, Darmstadt, Germany [6]. Agar powder (gel strength = 750 ± 50 g/cm^2^; melting point = 85 ± 5 °C) was purchased from Hayashi Pure Chemical Ind., Ltd., Osaka, Japan. TA was purchased from Kishida, Osaka, Japan. Pure water (Millipore, Burlington, MA, USA) with a resistivity of 18.2 MΩ cm was used as a solvent. All chemical reagents were used without further purification.

### 4.2. Methods

#### 4.2.1. Preparation of Hydrogels

Hetero-network hydrogels were prepared by adding PVA to an aqueous solution of TA, and the mixture was heated to 95 °C and stirred for 4 h. Subsequently, a certain amount of agar was added to the aqueous mixture of PVA and TA, and the blended solution was thoroughly stirred at 95 °C, then poured into a styrene square case, frozen at −15 °C for 24 h, and thawed at room temperature for 4 h. The amounts of PVA, agar, and TA used for different hydrogels are listed in Table 1. The hydrogels were labeled PVA, PA (mixture of PVA and agar), and PA-Tx (mixture of PVA, agar, and TA; x represents the TA concentration).

#### 4.2.2. Effects of Freezing Temperature, Freezing Time, and Number of F/T Cycles on the Mechanical Strength of PVA-Based Hydrogels

The following conditions were fixed in the present study:

1. To determine the freezing temperature, we conducted an experiment involving PVA only at −10 and −15 °C and finally set the freezing temperature to −15 °C.
2. For freezing and thawing durations, we adopted 24 h of freezing and 4 h of thawing.
3. We conducted experiments with 1, 3, and 5 F/T cycles, evaluated the mechanical properties, and examined the conditions resulting in the highest strength.

#### 4.2.3. FT-IR Analysis

The structural properties of PVA, PA, and PA-T1.0 were analyzed using FT-IR spectroscopy (FT/IR-4000, Jasco Inc., Tokyo, Japan). The analysis was conducted by subtracting the KBr background to investigate the possible molecular interactions between PVA, agar, and TA. All spectra were obtained in the wavelength range of 4000–500 cm^−1^ and were captured in transmission mode. The hydrogels samples were vacuum-dried before measurement.

#### 4.2.4. Mechanical Strength of the Hydrogels

The tensile strain–stress data were obtained using a tensile tester (Shimadzu EZ-LX, 100 N, Tokyo, Japan) at a strain rate of 50 mm/min. The hydrogel samples were constructed in the shape of rectangles and the height, width, and thickness of the samples were 15, 5, and 5 mm, respectively. We have opted to measure the hydrogels with a thickness of 5 mm in order to simulate native cartilage (4–5 mm) thickness [35]. The samples were attached to the lower and upper plates, and the load cell exerted on the hydrogel was stopped after the disruption of the specimen. For each sample, at least three measurements were performed, and the average result was noted.

#### 4.2.5. Crystallization Properties

The crystallization of PVA, PA, and PA-Tx was studied using XRD (Smart Lab, Rigaku, Tokyo, Japan). Liquid nitrogen was used to freeze the hydrogels, which were then freeze-dried in a freeze drier, FRD-mini (IWAKI, asahi techno glass, Tokyo, Japan) in vacuo at −50 °C for 2 days until all the solvents were sublimed. The XRD scan angles were in the range of 2–50°.

The degree of crystallinity (*Xc*) was calculated as follows:

(1) $$Xc=\left(\frac{Ac}{Ac+Aa}\right)\times 100$$

where *A*c is the area under the crystalline peaks, and *A*a + *A*c is the total area of amorphous and crystalline peaks.

#### 4.2.6. Swelling Ratio

Hydrogels with a thickness of 5 mm were cut into 15 mm × 10 mm pieces. The following equation was used to calculate the swelling ratios of PVA, PA, and PA-Tx:

(2) $$Swellingratio=\frac{\left(W_{t}-W_{d}\right)}{W_{d}}\times 100$$

where $W_{t}$ is the weight of the swollen or rehydrated gel measured at time *t*, and $W_{d}$ is the weight of the freeze-dried gel. Initially, the hydrogels were submerged in deionized water at 20 °C. After a predetermined time period, the swollen hydrogel samples were removed from the water bath and weighed. For accuracy, the samples were weighed three times.

#### 4.2.7. Water-Retaining Capacity

The water-retaining capacity (WRC) indicates the capacity of the gel structure to hold water. It was determined as follows: gel samples were sliced in half and centrifuged at 4800 rpm for 10, 20, and 30 min at 40 °C. After centrifugation, water was extracted from the hydrogel samples. The hydrogels were weighed before and after centrifugation, and the WRC of the PVA, PA, and PA-Tx hydrogels was calculated using the following equation [36].

(3) $$WRC(\%)=\frac{W_{a}}{W_{b}}\times 100$$

where *W_a_* and *W_b_* are the hydrogel weights after and before centrifugation, respectively.

#### 4.2.8. Hydrogel Adhesion

The hydrogel tensile adhesion was determined using a tensile tester (Shimadzu EZ-LX, 100 N, Tokyo, Japan) at a strain rate of 10 mm/min. Commercially accessible substrates such as rubber, glass, and aluminium were used. The substrates (25 mm × 75 mm × 2 mm) were cleaned with deionized water and ethanol and dried for use. To attach the substrate, a hydrogel of dimensions 5 mm × 19 mm × 5 mm was placed between two specimens and squeezed with a 100 g weight for 5 min. The hydrogels attached to the substrates were placed in the tensile tester and separated at a crosshead speed of 10 mm/min. The adhesive strength was determined by dividing the measured maximum load by the bonded area. Each sample was tested in triplicate.

## Figures and Tables

**Table 1:** Composition of PVA, PA, and PA-Tx hydrogels.

Hydrogels	PVA (wt%)	Agar (wt%)	TA (wt%)	Water (mL)
PVA	15	–	–	85
PA	15	1	–	85
PA-T0.25	15	1	0.25	85
PA-T0.5	15	1	0.5	85
PA-T0.75	15	1	0.75	85
PA-T1.0	15	1	1.0	85
PA-T1.5	15	1	1.5	85

**Table 2:** Crystallinity of PVA for different F/T cycles.

F/T Cycles	Crystallinity (%)
1	51.3
2	77.4
3	78.5

**Table 3:** Comparison of previously reported hydrogels with the current hydrogel in terms of their adhesion strength on different substrates.

Hydrogel [Reference]	Adhesion Strength (kPa)		
	Rubber	Glass	Aluminium
PVA–COOH/Polydopamine (PDA) [26]	150	–	–
PVA–Functionalized single-wall carbone nanotube–PDA [27]	8.3	9.2	–
PVA/Polyacrylamide interpenetrating network hydrogel [28]	70	70	–
PVA-Chitosan-black wattle tannin-Sodium chloride hydrogel [29]	2	3.4	–
Poly (vinyl alcohol) grafted with styryl pyridinium group (PVA-SbQ) and TEMPO-oxidized cellulose nanofibrils (CNF) [30]	30	36.5	–
Poly(1,3,4-dihdroxyphenyl-L-alanine acrylamide-meso-erythritol-polycaprolactone methacrylic anhydride) [31]	–	26.7	38.4
Nanogel-reinforced polyacrylamide [32]	–	74	78
Polydopamine/Carbomethyl cellulose/Polyacrylic acid (PDA/CMC/PAA) [33]	–	200 ± 25	340 ± 40
Current work (PA-T1.5)	60	232	227

## Data Availability

The data presented in this study are openly available in article.
